# Technology use in pediatric cardiovascular surgery intensive care nursing: a qualitative study

**DOI:** 10.1186/s12912-026-04867-7

**Published:** 2026-06-18

**Authors:** Aydın Nart, Meltem Aslan

**Affiliations:** 1 Department of Nursing, Faculty of Health Sciences, Istanbul Esenyurt University, Istanbul, Türkiye; 2 Department of Midwifery, Faculty of Health Sciences, Istanbul Esenyurt University, Istanbul, Türkiye

**Keywords:** Pediatric cardiovascular surgery intensive care, Cardiac surgery, Nursing, Technology, Qualitative research

## Abstract

**Background:**

The increasing integration of technology into intensive care settings has significantly influenced nursing practice, clinical decision-making, and care delivery processes. However, there is limited qualitative evidence exploring how nurses experience and interpret the use of technology, particularly in specialized settings such as pediatric cardiovascular surgery intensive care units.

**Aim:**

This study aimed to explore the experiences, perceptions, and evaluations of nurses regarding the use of technology in a pediatric cardiovascular surgery intensive care unit.

**Methods:**

This qualitative descriptive study was conducted with 24 nurses working in a pediatric cardiovascular surgery intensive care unit. Participants were recruited using snowball sampling. Data were collected through semi-structured, in-depth interviews lasting approximately 30–35 min. The interview guide was developed by the researchers based on relevant literature. Data were analyzed using reflexive thematic analysis as described by Braun and Clarke. The study was reported in accordance with the COREQ checklist, and trustworthiness was ensured using Lincoln and Guba’s criteria.

**Results:**

Four main themes were identified: (1) technology and care organization, (2) technological transformation in nursing practice, (3) institutional gaps in technological competence, and (4) multidimensional impact of technology. Technology was perceived as facilitating patient monitoring, supporting timely interventions, and contributing to more structured care processes. While participants indicated that technology may reduce physical workload, it was also associated with increased cognitive demands. Nurses reported that existing training opportunities were limited and emphasized the need for more structured and practice-based educational support.

**Conclusion:**

Technology is perceived as an integral component of care in pediatric cardiovascular surgery intensive care settings, influencing both clinical processes and nursing practice. However, the findings suggest that its effective and safe use depends on strengthened training programs, institutional support, and attention to cognitive and ethical dimensions of care.

**Supplementary Information:**

The online version contains supplementary material available at 10.1186/s12912-026-04867-7.

## Introduction

Intensive care units (ICUs) are complex environments where advanced technologies are utilized and life-threatening conditions are managed. In these settings, nurses are responsible for continuously monitoring multiple physiological parameters simultaneously and making rapid and accurate clinical decisions. In recent years, with the increasing digitalization of healthcare, particularly artificial intelligence–based applications have significantly influenced care processes by enabling continuous analysis of patient data and the early detection of clinical deterioration, thereby enhancing the effectiveness of care [1, 2].

Intensive care technologies are transforming not only clinical outcomes but also nursing workflows and the organisation of care. Whilst monitoring systems, ventilators and electronic records promote standardisation, they also bring with them challenges such as new skill requirements, increased cognitive load and the need to adapt to technology [3]. Furthermore, usability issues, repetitive data entry and inadequate training can negatively impact nurses’ job satisfaction and system effectiveness; the lack of transparency in artificial intelligence systems, meanwhile, can limit clinicians’ trust [1, 4].

However, intensive care nursing is not solely technology-driven; communication with patients and the human aspect of care remain of paramount importance. Non-verbal communication, in particular, plays a critical role in the delivery of care in intensive care units [5]. This highlights the need for a balanced approach between the use of technology and patient-centred care.

The literature indicates that there are few studies examining intensive care nurses’ experiences with technology. In particular, there is a notable lack of qualitative research focusing on the effects of technology on workflow, workload and professional roles [2, 6]. This shortfall is even more pronounced in highly specialised fields such as paediatric intensive care.

Although previous studies have examined technology use and digitalization in intensive care nursing, most research has focused on general adult ICU settings, technology acceptance, or quantitative evaluations of workflow efficiency. Limited qualitative evidence exists regarding how nurses interpret and negotiate technology use within highly specialized pediatric cardiovascular surgery intensive care environments, where care involves complex hemodynamic monitoring, high-risk medication management, family-centered communication, and ethically sensitive decision-making. In pediatric cardiovascular intensive care settings, nurses must simultaneously manage technological surveillance, rapid clinical deterioration, and the emotional vulnerability of children and families. These context-specific demands may shape the meaning and impact of technology in ways that differ from general intensive care environments. Therefore, this study seeks to provide an in-depth qualitative understanding of how technology reshapes nursing practice, professional responsibility, cognitive workload, and relational aspects of care within pediatric cardiovascular surgery intensive care nursing.

This study was conceptually informed by sociotechnical perspectives, which emphasize the dynamic interaction between healthcare professionals, technological systems, clinical workflows, and organizational structures. From this perspective, technology is understood not merely as a technical tool, but as a factor shaping professional roles, communication patterns, cognitive processes, and patient care practices within intensive care environments. This perspective also informed the interpretation of how nurses negotiate the balance between technological surveillance and human-centered pediatric care.

## Methods

### Study design

This study was conducted using a qualitative descriptive design informed by a pragmatic and experiential orientation to explore nurses’ experiences regarding technology use in a pediatric cardiovascular surgery intensive care unit. Qualitative description was considered appropriate because the study aimed to provide a practice-oriented and clinically grounded understanding of participants’ experiences rather than generate abstract theory [7].

To analyze patterns of meaning across participants’ accounts, reflexive thematic analysis as conceptualized by Braun and Clarke was employed. Although qualitative description is generally associated with low-inference interpretations, reflexive thematic analysis enabled the researchers to move beyond surface-level descriptions and explore how nurses interpreted the influence of technology on professional roles, cognitive workload, patient safety, and care relationships within this specialized clinical context. The analysis was guided by an interpretive–descriptive epistemological stance, acknowledging the active role of researchers in meaning construction while remaining closely connected to participants’ experiential accounts.

The study was reported in accordance with the Consolidated Criteria for Reporting Qualitative Research (COREQ) checklist to ensure transparency and methodological rigor [8].

### Participants and sampling method

The study sample consisted of 24 nurses working in a pediatric cardiovascular surgery (CVS) intensive care unit who voluntarily agreed to participate. A snowball sampling method was used to recruit participants [9], whereby nurses recommended colleagues who met the inclusion criteria and had relevant experience in technology-intensive pediatric critical care settings. This sampling approach was considered appropriate because pediatric cardiovascular surgery intensive care nurses represent a relatively specialized clinical group with demanding shift schedules and specific technological expertise, making access to experienced participants more feasible through professional networks. The sample size was determined based on the principle of data saturation, and data collection was concluded when no new themes emerged [10]. The inclusion criteria were defined as actively working in the unit, direct involvement in the use of technology, having at least six months of professional experience, and providing voluntary participation (including consent for audio recording). To ensure confidentiality, participants were coded as N1–N24.

### Data collection process

Data were collected through semi-structured, in-depth interviews [11]. The semi-structured interview guide was developed by the researchers specifically for this study based on the relevant literature (Table 1). The English version of the interview guide is provided as Supplementary File 1**.**

**Table 1:** Interview Guide

• Can you describe your experience of using technology in the pediatric cardiovascular surgery intensive care unit?
• What types of technologies do you use most frequently in your daily practice?
• How does technology influence patient care and clinical decision-making in your unit?
• In what ways does technology affect your daily work and workload?
• How does working with technology influence your professional competence or job satisfaction?
• Can you describe your experiences with training and support related to technology use?
• How does technology influence patient safety, communication, or ethical aspects of care?
• How do you think technology will shape the future of intensive care nursing?

Interviews were conducted at times convenient for the participants and in settings that ensured privacy. Each interview lasted approximately 30–35 min and was audio-recorded with participants’ consent. During the interviews, open-ended questions were used to enable participants to express their experiences in detail, and probing questions were asked when necessary to obtain deeper insights.

### Reflexivity

Interviews were conducted by a researcher with experience in qualitative research and pediatric surgical nursing. Reflexive field notes were maintained throughout the study to enhance analytical rigor and minimize potential bias.

### Data management and transcription process

Audio recordings obtained from the interviews were transferred to secure digital environments and transcribed using Transkriptor software. The transcripts were checked by the researchers for accuracy. The finalized transcripts were then imported into MAXQDA software for data analysis.

### Data analysis

Data were analyzed using the six-phase reflexive thematic analysis approach described by Braun and Clarke [12].

The analysis included:

1. Familiarization with data.
2. Generating initial codes.
3. Searching for themes.
4. Reviewing themes.
5. Defining and naming themes.
6. Producing the report.

Coding was conducted inductively. During the analytic process, initial semantic codes were generated from participants’ narratives and compared across transcripts to identify recurring patterns of meaning. Related codes were then clustered into broader interpretive categories and progressively refined into themes and subthemes through iterative analysis. For example, codes related to “alarm monitoring,” “continuous screen attention,” and “data interpretation” were grouped under the broader subtheme of cognitive workload. Differences in coding and interpretation between researchers were discussed during reflexive analytic meetings until a shared understanding was achieved. Analytic memos and coding comparisons were also used to document how themes evolved throughout the analytic process.

### Scientific validity and reliability

Trustworthiness was ensured according to Lincoln and Guba’s criteria of credibility, transferability, dependability, and confirmability [13]. Credibility was strengthened through prolonged engagement with the data, repeated reading of transcripts, peer debriefing between researchers, and the use of rich participant quotations to support analytical interpretations. In addition, preliminary thematic interpretations were reviewed among the research team to enhance interpretive consistency. In addition, selected participants were consulted during the later stages of analysis to confirm whether the thematic interpretations reflected their clinical experiences, thereby strengthening credibility through participant validation. Transferability was supported by providing detailed descriptions of the clinical setting, participant characteristics, and data collection procedures to allow readers to evaluate the applicability of findings to similar contexts. Dependability was enhanced through maintaining a transparent audit trail documenting interview procedures, coding decisions, theme development, and analytic reflections throughout the study process. Confirmability was supported through reflexive field notes, analytic memos, and continuous reflection on potential researcher assumptions during data interpretation.

To further strengthen methodological rigor, trustworthiness strategies were implemented throughout different stages of the study rather than being applied only after data analysis. During data collection, the interviewer used probing questions and maintained reflexive field notes after each interview. During transcription and data preparation, all transcripts were checked against audio recordings for accuracy. During analysis, researchers independently reviewed transcripts, discussed coding decisions, and documented changes in codes and themes through analytic memos. Peer debriefing was conducted during theme development to question emerging interpretations and reduce the risk of individual researcher bias. These procedures provided a transparent audit trail demonstrating how interpretations were developed from raw data to final themes.

Additionally, peer debriefing and expert review were used to strengthen methodological rigor.

## Results

In this study, the experiences and perceptions of pediatric cardiovascular surgery (CVS) intensive care nurses regarding the use of technology were examined under four main themes and their subthemes. Participants were coded as N1–N24. The findings indicate that technology is not merely a technical tool; rather, it is a multidimensional factor that transforms the structure of care, workload, professional experience, and patient safety.

### Sociodemographic characteristics

A total of 24 nurses took part in the study. Half of the participants were in the 25–34 age group (50.0%, *n* = 12), whilst the others were in the 35–44 (25.0%, *n* = 6), 18–24 (16.7%, *n* = 4) and 45–54 (8.3%, *n* = 2) age groups. 66.7% of the participants (*n* = 16) were female and 33.3% (*n* = 8) were male; 58.3% (*n* = 14) were single and 41.7% (*n* = 10) were married.

In terms of professional experience, the majority of participants had 4–6 years’ experience (41.7%, *n* = 10), followed by those with 1–3 years and 7–10 years (25.0%, *n* = 6); 8.3% (*n* = 2) had 11–15 years’ experience. Pediatric cardiac surgery intensive care experience was predominantly 4–6 years (58.3%, *n* = 14), with the remainder falling within the 1–3 year (25.0%, *n* = 6) and 7–10 year (16.7%, *n* = 4) ranges. 58.3% of participants (*n* = 14) hold a bachelor’s degree, 25.0% (*n* = 6) a master’s degree, 8.3% (*n* = 2) a PhD, and 8.3% (*n* = 2) an associate degree.

58.3% of nurses (*n* = 14) work 16-hour shifts, whilst 41.7% (*n* = 10) work 8-hour shifts. In terms of technological self-efficacy, 41.7% of participants (*n* = 10) rated themselves as highly competent, 33.3% (*n* = 8) as competent, and 25.0% (*n* = 6) as moderately competent. 66.7% of participants (*n* = 16) have received training or certification in technology, whilst 83.3% (*n* = 20) stated that they would like to undertake further training.

### Qualitative findings

#### Theme 1. Technology and care organization

This theme demonstrates that technology is positioned not merely as an auxiliary tool in paediatric cardiac and vascular intensive care, but as a fundamental element that shapes the structure of care. Participants’ statements reveal that a broad technological infrastructure—ranging from ventilators to ECMO, and from infusion pumps to central monitoring systems—has become an integral part of care. Nurses emphasised that these devices not only facilitate patient monitoring but also standardise care processes, accelerate decision-making, and ensure the sustainability of life support for critically ill patients.

##### Subtheme 1.1. Intensive care technologies

Participants’ narratives indicated that the pediatric cardiovascular surgery (CVS) intensive care unit is a technology-driven care environment. Ventilators, monitoring systems, infusion and syringe pumps, extracorporeal membrane oxygenation (ECMO), dialysis/continuous renal replacement therapy (CRRT) systems, and electronic health record infrastructures were highlighted. These findings demonstrate that technology is not a secondary component, but a fundamental element that ensures the sustainability of care. Nurses described these systems as “critical” and “indispensable,” indicating that technology has evolved beyond a supportive tool to become a clinical ecosystem shaping the care environment. The importance of invasive monitoring and life-support devices was particularly emphasized in the management of unstable patients following cardiac surgery.

***N5:**** “In our unit*,* the most commonly used technologies include mechanical ventilators*,* bedside monitoring systems*,* and infusion pumps. These systems are indispensable*,* particularly for ensuring hemodynamic monitoring in patients following cardiac surgery.”*

***N18: ****“Monitors*,* ventilators*,* infusion and syringe pumps*,* and when necessary*,* ECMO and dialysis devices are the technological tools we use most frequently. These systems make real-time patient monitoring much safer and significantly ease our workload.”*

***N21:**** “ECMO*,* dialysis*,* and advanced life-support devices are critical for us. In addition*,* central monitoring systems that allow us to track patient data in real time are an essential part of care.”*

##### Subtheme 1.2. Early monitoring and data-driven care

Participants identified the most significant contribution of technology as the early detection of patient deterioration and the acquisition of objective data. In this context, technology transforms nursing care from an observation-based approach into a more measurable, traceable, and evidence-informed practice. In particular, monitoring systems, infusion pumps, and ventilators were emphasized as essential tools for the early identification of complications and timely intervention. These findings suggest that nurses perceive technology not merely as a facilitator of tasks, but as a key component of quality assurance that directly influences patient safety and care quality. Considering the sensitivity of parameters such as dosage, fluid balance, and hemodynamic status in pediatric patients, technological systems appear to enhance accuracy, continuity, and safety in care delivery.

***N5:**** “Thanks to technological devices*,* we are able to monitor patients continuously and in real time. Early detection of hemodynamic changes accelerates intervention*,* which provides a significant advantage for the patient. It also makes our work much easier. These devices allow complications to be identified early*,* enabling timely intervention before problems escalate.”*

***N16:**** “It significantly reduces the margin of error in the care process. For example*,* infusion pumps allow medications to be administered in a much more controlled manner*,* which increases safety*,* especially in pediatric patients. As these are high-technology devices*,* they also positively influence patient morbidity and mortality rates.”*

***N20:**** “I believe it improves the quality of care. Because technological devices provide more objective measurements*,* we make clinical decisions based on more reliable data.”*

##### Subtheme 1.3. Transition to digital and automated processes

Interviews revealed that the use of technology has led to a structural transformation in nursing procedures. Participants reported that many practices previously performed manually are now carried out through devices and digital systems. In particular, processes such as fluid and medication monitoring, vital sign tracking, documentation, and care planning have shifted from manual execution to system-based management. While this transformation has increased the speed and standardization of care and strengthened institutional documentation, it has also reshaped the role of nurses from direct task performers to monitors and interpreters of technological systems.

***N13:**** “We have gradually transitioned from manual processes to more automated systems. Our work has become increasingly system-based rather than manual*,* with a greater emphasis on technological tasks.”*

***N18:**** “There has been a significant change in documentation processes. Many data that we previously recorded on paper are now entered into electronic systems*,* which has improved organization.”*

***N19:**** “Care planning has become more systematic. Based on the data obtained from devices*,* we are able to make clearer and faster decisions regarding patient care.”*

#### Theme 2. Technological transformation in nursing

This theme reflects the impact of technology on nurses’ daily work experience. Participants noted that technology facilitates care processes, systematises workflows and, in some respects, enhances job satisfaction. However, it is also evident that the use of technology does not entirely eliminate the workload; rather, whilst reducing physical strain, it increases cognitive, monitoring and technical demands. Consequently, technology is not a linear factor that reduces the nursing workload; it is a factor that reshapes the nature of the work.

##### Subtheme 2.1. Supportive use of technology

Participants reported that the use of technology enables care to be delivered in a more controlled, organized, and safe manner, thereby increasing job satisfaction. It was emphasized that technology facilitates time management, allows more effective monitoring of multiple patients simultaneously, and reduces the risk of errors. This, in turn, contributes to nurses feeling more competent and professional.

Moreover, technology not only provides physical convenience but also fosters a sense of psychological security, supporting clinical decisions based on more reliable data and enhancing professional confidence and job satisfaction.

***N15:**** “Technology significantly facilitates my work*,* which increases my job satisfaction. Especially in intensive care*,* we have to monitor multiple patients simultaneously*,* and with monitors and other devices*,* we can track patients’ conditions more quickly and safely. This reduces my workload and helps me feel more in control and secure.”*

***N17:**** “The use of technology increases my job satisfaction because it makes time management easier. Tasks that we previously performed manually took much longer*,* whereas now we can proceed more quickly with the help of devices. This allows me to allocate more time to patients and increases my overall satisfaction with my work.”*

***N20:**** “Using technology makes me feel more professionally competent. Working with a lower risk of error and basing my clinical decisions on more reliable data increases my confidence in my work*,* which is reflected in my job satisfaction.”*

##### Subtheme 2.2. Cognitive load and workload

The data indicate that technology does not reduce workload in a unidirectional manner but rather transforms it. While automation and the reduction of manual tasks alleviate physical workload, responsibilities such as device monitoring, alarm management, data entry and interpretation, and troubleshooting increase cognitive load. These findings suggest that technology does not simply make work easier; instead, it redistributes workload from physical labor to cognitive monitoring and technical responsibility.

***N16:**** “Technology reduces workload in many ways*,* but it does not eliminate it entirely. Some tasks become faster*,* but monitoring devices*,* managing alarms*,* and entering data bring additional responsibilities. I feel that the workload has changed its form.”*

***N20:**** “I think it reduces physical workload but increases mental workload. Constant screen monitoring*,* interpreting data*,* and anticipating potential errors require sustained attention.”*

***N22:**** “I believe it transforms rather than reduces workload. We perform fewer manual tasks*,* but we engage more in managing technology. This creates a different domain of responsibility.”*

#### Theme 3. Institutional gaps in technological competence

This theme indicates that nurses’ knowledge and skills regarding the use of technology have largely developed through fragmented training processes that are provided on an ad-hoc basis and are not fully institutionalised. Participants noted that, in most cases, brief introductions are provided when a new device arrives, basic information is given during the induction process, or device-specific training is received from company representatives. This suggests that, in an intensive care setting utilising advanced technology, training processes often remain reactive.

##### Subtheme 3.1. Fragmented training and clinical learning

Participants’ statements indicated that technology-related training is often conducted in response to specific situations—such as the introduction of new devices, the occurrence of technical problems, or orientation needs—rather than through comprehensive institutional programs. Consequently, learning tends to occur within the workflow through trial-and-error and brief instructions, rather than through structured pedagogical processes.

This finding suggests that the development of technological competence in technology-intensive clinical settings is not systematically ensured. In high-risk environments such as pediatric cardiovascular surgery (CVS) intensive care units, reliance solely on experience-based learning appears to be insufficient.

***N3:*** *“Honestly, I have not received comprehensive training on technology. Usually, brief introductions are provided when a new device arrives, and we mostly learn through practice.”*

***N17:*** *“We mostly received device-specific training from company representatives. When a new device is introduced, they explain how to use it, but these trainings are generally short and superficial.”*

***N22:*** *“Training is usually need-based. Information is provided when a problem occurs or when a new technology is introduced, but I cannot say that there is a systematic and continuous training process.”*

##### Subtheme 3.2. Inadequate training and need for development

Participants considered the existing training insufficient in terms of content, practical application, and continuity. They emphasized the need for more comprehensive education, particularly in advanced device use, complication management, and adaptation to new technologies. The findings indicate that training is often limited to basic operational knowledge and does not adequately prepare nurses for clinically complex situations. This suggests that competence in technology-intensive care environments is not solely related to device operation, but also involves skills such as problem-solving, clinical decision-making, and managing unexpected situations.

***N4:*** *“Honestly*,* I do not think it is sufficient. It mostly remains at a basic level; more comprehensive and practice-based training is needed*,* especially for advanced device use.”*

***N22:*** *“Some theoretical information is provided*,* but it remains insufficient in practice. In an environment like intensive care*,* I believe there should be more hands-on training.”*

***N24:*** *“Overall*,* it is not sufficient. Especially in a specialized area such as pediatric CVS intensive care*,* more specific and advanced-level training should be provided.”*

##### Subtheme 3.3. Structured and sustainable development

Participants not only emphasized the inadequacy of existing training but also articulated how it should be improved. Their recommendations included regularly scheduled training programs, simulation-based learning, certified educational programs, mentorship, and flexible learning models adapted to shift work. These findings indicate that nurses are not passive recipients, but active agents who can identify their educational needs and propose solutions for professional development.

***N5:*** *“I believe that training should be conducted in a more planned and regular manner. Especially if hands-on training is increased*,* learning becomes more lasting and we can work more confidently in clinical settings.”*

***N9:*** *“Training should not be limited to theory; simulation-based education should also be implemented. Experiencing critical situations that we may encounter in intensive care beforehand would be very beneficial.”*

***N19:*** *“I think certified training programs should be increased. Training focused on specialization*,* particularly in areas such as ECMO and ventilator management*,* would be very useful.”*

#### Theme 4. Multidimensional impact of technology

This theme demonstrates that technology shapes perceptions not only of clinical processes but also of patient safety, communication with families, data privacy, ethical use and the future of the profession. Whilst participants described technology as a factor that builds trust, enhances the quality of care and offers hope for the future, they also highlighted risk areas such as privacy, user error, lack of legal knowledge and over-reliance on technology.

##### Subtheme 4.1. Safety and proper use

Participants emphasized that monitoring systems, infusion and syringe pumps, ventilators, ECMO/CRRT, and alarm systems are critical for patient safety, particularly highlighting early warning capabilities, dosage precision, real-time monitoring, and accurate documentation. However, some nurses stressed that safety is not solely dependent on the devices themselves, but rather on their correct use and adequate training. This finding suggests that technology alone does not ensure safety; instead, safety emerges through the interaction between humans and technology—an aspect that is particularly crucial in high-risk settings such as pediatric intensive care units.

***N15:*** *“I believe that bedside monitoring systems are particularly critical for patient safety. Being able to continuously monitor parameters such as heart rhythm*,* oxygen saturation*,* and blood pressure allows us to detect potential deterioration early and intervene promptly.”*

***N11:*** *“In my opinion*,* infusion and syringe pumps are among the most effective technologies. Medication dosages in pediatric patients are highly sensitive*,* and these devices help prevent dosing errors*,* directly improving patient safety.”*

***N22:*** *“In general*,* all technologies complement each other*,* but I think pumps with automatic dose adjustment and monitors that provide continuous data are the most effective in enhancing patient safety.”*

##### Subtheme 4.2. Technology and family communication

Participants’ narratives indicated that technology has a dual impact on communication with patients’ families. On one hand, families tend to feel more reassured knowing that their children are being continuously monitored through technological devices. In addition, access to real-time data enables nurses to provide clearer and more up-to-date information.

On the other hand, an excessive focus on technological devices may lead families to become anxious about minor changes and may reduce face-to-face interaction. These findings suggest that communication mediated by technology is not only facilitated but also requires interpretation, explanation, and emotional support, representing a more complex form of interaction.

***N15:*** *“Technology generally facilitates communication with patients and their families. Especially the ability to quickly access laboratory results and clinical data through the system allows families to obtain information more rapidly. This reduces their anxiety to some extent and increases their trust in us.”*

***N18:*** *“Although there are positive aspects*,* there can also be negative effects. Families may focus too much on the devices and become anxious even about minor changes. In such cases*,* we need to provide more explanations.”*

***N21:*** *“Sometimes I think technology can make communication more distant. While focusing on devices*,* there may be less time for direct*,* face-to-face interaction with families.”*

##### Subtheme 4.3. Lack of ethical and legal knowledge

Participants generally considered electronic health records to be secure; however, they reported having limited knowledge regarding ethical and legal aspects. While access restrictions, username–password systems, and traceability increased their sense of security, practices such as password sharing, leaving screens open, user-related errors, and cybersecurity risks were identified as potential threats.

Although participants stated that they had not experienced serious ethical issues, they highlighted overreliance on technology, potential breaches of confidentiality, and uncertainties regarding data use as areas of concern. A lack of knowledge about legal regulations and the need for in-service training were emphasized. These findings suggest that ethical and legal competence should be developed alongside technological competence.

***N12:*** *“I believe that the confidentiality of electronic health records is generally well protected. Systems require login with a username and password*,* and everyone can only access patients within their own authorization scope. This provides reassurance in terms of protecting patient information.”*

***N19:*** *“The most concerning ethical issue is who uses patient information and for what purpose. I think there should be clearer boundaries and stricter monitoring in this regard.”*

***N22:*** *“I believe my knowledge on this topic is limited. Legal responsibilities should be explained more clearly*,* and regular updates about current regulations should be provided.”*

##### Subtheme 4.4. Future clinical technologies

Participants generally evaluated the future as positive and promising, emphasizing technologies such as artificial intelligence–supported early warning systems, integrated monitoring platforms, wireless monitoring, robotic assistance, automated digital documentation, tele-intensive care, and personalized sensors. These findings suggest that nurses are not only users of technology but also actors capable of anticipating the future of care environments.

However, participants also highlighted that the increasing role of technology should not overshadow nurses’ clinical knowledge. Their vision of the future reflects not only the expectation of more advanced technologies but also the need for appropriate integration and human-centered use.

***N6:*** *“I believe that artificial intelligence–supported systems will become more widely used. Especially early warning systems that predict patient deterioration will strengthen nursing care and enable faster interventions.”*

***N17:*** *“I think that smart patient monitoring systems*,* where all devices are integrated and data are automatically analyzed to provide recommendations*,* will significantly facilitate the care process.”*

***N23:*** *“With the development of remote monitoring and tele-intensive care systems*,* it will be possible to follow patients even after discharge from intensive care. This could improve continuity of care and reduce readmissions.”*

## Discussion

This study provides an in-depth understanding of nurses’ experiences with technology use in a pediatric cardiovascular surgery intensive care unit, highlighting its perceived impact on clinical care processes and nursing practice. The findings are largely consistent with existing literature and offer additional insight into technology use in pediatric intensive care settings, particularly in relation to cognitive workload, evolving professional roles, and institutional support needs.

### The integration of technology into care

In this study, nurses described technology as an integral component of care, supporting clinical monitoring, early intervention, and the standardization of care processes. This finding aligns with the data-intensive nature of intensive care environments and previous research demonstrating that machine learning–based systems can predict early deterioration and support clinical decision-making [14]. Similarly, early warning systems have been shown to predict critical events in advance, highlighting the contribution of technology to patient safety [15]. Artificial intelligence applications have also been reported to optimize monitoring, data analysis, and clinical decision support processes in intensive care nursing [16]. Together, these findings suggest that technology is increasingly embedded within care processes rather than functioning solely as a supportive tool.

In pediatric cardiovascular surgery intensive care units, technology appears to function not only as a monitoring tool but also as part of a broader sociotechnical care system shaping clinical decision-making, workflow organization, and interdisciplinary coordination. Unlike adult intensive care settings, pediatric cardiovascular care involves fragile patients, rapidly changing hemodynamic parameters, and highly sensitive medication dosages. Therefore, nurses described technology as essential for continuous surveillance and early intervention. However, the findings also suggest that patient safety depends not only on technology itself, but on nurses’ ability to interpret and integrate technological data into clinical judgment. Furthermore, the PICU environment introduces emotional and ethical complexities in which nurses must balance technological monitoring with family-centered communication and human-centered care.

### The transformation of nursing experience

Technology was perceived as both facilitating and complicating nursing practice. While it supported efficiency and clinical reasoning, it also increased cognitive workload and responsibility. This finding is consistent with studies indicating that digital technologies and artificial intelligence may automate certain tasks while simultaneously increasing nurses’ responsibilities in clinical decision-making processes [17]. Clinical decision-making in intensive care is inherently complex and cognitively demanding [18], and previous research has shown that positive attitudes toward artificial intelligence are associated with increased clinical reasoning and self-efficacy among nurses [19]. Similarly, favorable attitudes toward technology have been linked to enhanced professional self-efficacy in intensive care settings [20]. These findings suggest a shift from predominantly physical workload toward more cognitively demanding forms of nursing practice.

The findings suggest that technology transforms nursing work beyond reducing physical workload by increasing responsibilities related to continuous monitoring, alarm interpretation, and rapid clinical decision-making. This shift may be particularly significant in pediatric cardiovascular intensive care units, where minor physiological changes can quickly become life-threatening. Although technology supports patient monitoring and safety, continuous exposure to alarms and multiple monitoring systems may contribute to cognitive fatigue and decision overload. Accordingly, nursing expertise in PICU settings increasingly depends on integrating technological data with clinical judgment and relational care, reflecting a transition toward more cognitively demanding nursing practice.

### Institutional gaps in technological competence

Another important finding relates to institutional gaps in technological competence. Nurses reported insufficient training and a need for continuous professional development. This finding is consistent with previous studies indicating that nurses may have limited knowledge of emerging technologies and a high need for education in this area [21]. The effective use of technology depends not only on individual competencies but also on institutional training programs and organizational support mechanisms [20]. Furthermore, it has been emphasized that technology-based care processes should be structured through systematic and evidence-based approaches [22]. These findings suggest that technological competence should be supported at both individual and organizational levels.

### Safety, ethics, and the human factor

In this study, technology was perceived as enhancing patient safety while also raising ethical and professional considerations. The literature similarly indicates that artificial intelligence provides advantages in clinical decision support but also introduces challenges such as algorithmic bias, lack of transparency, and trust-related concerns [23]. Studies involving intensive care nurses highlight the importance of trust, collaboration, and communication in technology use, as well as the need to preserve patient autonomy [17]. In addition, technology has been reported to influence nurses’ subjective experiences in both positive and negative ways [24]. These findings suggest that technology in nursing practice extends beyond technical functionality and involves ethical, relational, and professional dimensions.

Ethical considerations may be more complex in pediatric intensive care settings, where critically ill children depend heavily on both technological support and family-based decision-making. Nurses emphasized concerns related not only to patient safety and confidentiality, but also excessive dependence on technology and the weakening of direct human interaction. In pediatric cardiovascular surgery intensive care units, technological monitoring may dominate clinical attention despite children and families continuing to require emotional support and compassionate communication. Moreover, because pediatric medication dosages and hemodynamic parameters are highly sensitive, technological or user-related errors may have serious consequences.

These findings suggest that technology in nursing practice extends beyond technical functionality and also involves ethical, relational, and professional dimensions. Therefore, technological competence in PICU settings should include ethical awareness, communication skills, and critical clinical judgment alongside technical proficiency.

### Pediatric intensive care context

The pediatric intensive care context increases both the benefits and challenges associated with technology use due to the vulnerability of the patient population and the complexity of care processes. In pediatric cardiovascular surgery intensive care units, the need for precise medication titration, continuous hemodynamic monitoring, and rapid responses to sudden clinical changes increases nurses’ dependence on technological systems while also making the consequences of technological failure or delayed interpretation more critical.

In addition, within these family-centered care settings, nurses are required not only to manage technological systems but also to support families experiencing anxiety and uncertainty. Participants indicated that technology may be both reassuring and overwhelming for families.

These findings suggest that technology use in pediatric intensive care extends beyond technical competence and represents a multidimensional process requiring clinical judgment, communication skills, and ethical sensitivity.

### Implications for nursing practice

From a clinical perspective, these findings highlight the need for structured and practice-oriented training programs, clear clinical protocols, and institutional support systems to ensure the effective and safe use of technology in intensive care settings. Strengthening nurses’ technological competence may contribute to improved patient safety, more effective clinical decision-making, and enhanced quality of care.

Overall, the findings suggest that technology in pediatric cardiovascular surgery intensive care should not be conceptualized merely as equipment supporting nursing tasks. Instead, technology appears to restructure how nurses observe patients, interpret clinical deterioration, communicate with families, and assume professional responsibility. This broader interpretation moves beyond a purely instrumental understanding of technology and highlights the sociotechnical complexity of contemporary pediatric intensive care nursing.

## Conclusion

This study suggests that technology in pediatric cardiovascular surgery intensive care is experienced not merely as a clinical support tool, but as a structural component that reshapes nursing surveillance, cognitive responsibility, and care organization. The findings indicate that technological systems transform nursing work by shifting responsibilities from predominantly physical tasks toward continuous cognitive monitoring, interpretation, and technological coordination. At the same time, nurses identified important challenges related to ethical awareness, institutional preparedness, and the sustainability of technology-oriented competence. These findings highlight that the effective integration of technology in pediatric intensive care depends not only on technical infrastructure, but also on continuous professional development, organizational support, and the preservation of human-centered care within increasingly technology-mediated clinical environments.

## Limitations

This study has several limitations. The use of snowball sampling may have introduced selection bias, as participants could have shared similar perspectives. In addition, the data were based on self-reported experiences, which may be subject to individual interpretation and recall bias. Furthermore, as a qualitative study, the findings are not intended to be generalizable but rather to provide an in-depth understanding of nurses’ experiences within this specific context.

## Supplementary Information

Below is the link to the electronic supplementary material.

Supplementary Material 1

## Data Availability

The datasets used and/or analyzed during the current study are available from the corresponding author on reasonable request.
