# Supplementary material for: Technology use in pediatric cardiovascular surgery intensive care nursing: a qualitative study
Source: BMC Nurs. 2026 Jun 18;25:747. doi: 10.1186/s12912-026-04867-7 (PMC13501550; doi:10.1186/s12912-026-04867-7)
Supplement: Supplementary file 1 [file 12912_2026_4867_MOESM1_ESM.pdf]

Table 1: Interview Guide

• Can you describe your experience of using technology in the pediatric cardiovascular surgery intensive care unit?
• What types of technologies do you use most frequently in your daily practice?
• How does technology influence patient care and clinical decision-making in your unit?
• In what ways does technology affect your daily work and workload?
• How does working with technology influence your professional competence or job satisfaction?
• Can you describe your experiences with training and support related to technology use?
• How does technology influence patient safety, communication, or ethical aspects of care?
• How do you think technology will shape the future of intensive care nursing?
